# The role of probiotic therapy on clinical parameters and human immune response in peri-implant diseases: a systematic review and meta-analysis of randomized clinical studies

**DOI:** 10.3389/fimmu.2024.1371072

**Published:** 2024-04-15

**Authors:** Nansi López-Valverde, Antonio López-Valverde, José Antonio Blanco Rueda

**Affiliations:** ^1^ Department of Medicine and Medical Specialties, Faculty of Health Sciences, University of Alcalá de Henares, Madrid, Spain; ^2^ Department of Surgery, Instituto de Investigación Biomédica de Salamanca (IBSAL), University of Salamanca, Salamanca, Spain

**Keywords:** probiotic, prebiotic, peri-implant disease, mucositis, peri-implantitis, inmune-response, randomized clinical trial

## Abstract

**Background:**

Peri-implant diseases (peri-implant mucositis and peri-implantitis) are pathologies of an infectious-inflammatory nature of the mucosa around dental implants. Probiotics are microorganisms that regulate host immunomodulation and have shown positive results in the treatment of peri-implant diseases. The objective of the systematic review and meta-analysis was to evaluate the efficacy of probiotics in the treatment of peri-implant oral diseases.

**Methods:**

According to the PRISMA guidelines, the research question was established: Are probiotics able to favorably modify clinical and immunological biomarkers determinants of peri-implant pathologies? and an electronic search of the databases MEDLINE/PubMed, Embase, Cochrane Central, Web of Science, (until December 2023) was performed. Inclusion criteria were established for intervention studies (RCTs), according to the PICOs strategy in subjects with peri-implant pathology (participants), treated with probiotics (intervention) compared to patients with conventional treatment or placebo (control) and evaluating the response to treatment (outcomes). Results- 1723 studies were obtained and 10 were selected. Risk of bias was assessed using the Cochrane Risk of Bias Tool and methodological quality using the Joanna Briggs Institute for RCTs. Two meta-analyses were performed, one to evaluate probiotics in mucositis and one for peri-implantitis. All subgroups were homogeneous (I^2 = ^0%), except in the analysis of IL-6 in mucositis (I^2 = ^65%). The overall effect was favorable to the experimental group in both pathologies. The analysis of the studies grouped in peri-implantitis showed a tendency to significance (p=0.09).

**Conclusion:**

The use of probiotics, as basic or complementary treatment of peri-implant diseases, showed a statistically significant trend, but well-designed studies are warranted to validate the efficacy of these products in peri-implant pathologies.

## 1 Introduction

Peri-implant oral diseases (peri-implant mucositis and peri-implantitis) are a group of pathologies of an infectious nature, which describe, in the case of peri-implant mucositis, an inflammatory lesion of the mucosa surrounding a dental implant, while in peri-implantitis the supporting bone is affected. The most important parameter for the diagnosis of peri-implant mucositis is bleeding on probing, with a gentle pressure of less than 0.25 N; however, in peri-implantitis alterations appear at the crestal bone level and the presence of purulent fluid in the affected areas is frequent (1). This last aspect is the reason why peri-implantitis, unlike mucositis, is considered an irreversible pathology (2).

The consensus of the Sixth European Workshop on Periodontology (3) reported an incidence of mucositis in dental implants of up to 80% and between 28% and 56% of peri-implantitis, despite the controversy surrounding these high figures (4). This leads to failure of the implanted device, with consequent economic damage (5).

Peri-implantitis is considered a polymicrobial infection associated with *Staphylococcus epidermidis* and specific gram-negative periodontopathogens, such as *Porphyromonas gingivalis*, *Tannerella forsythia*, *Fusobacterium nucleatum* and *Porphyromona intermedia* (6) and although the mechanism of the microbial interaction is not precisely known, according to recent sequencing studies, a great diversity of bacterial species are considered responsible (7).

The term probiotic is used to define health-promoting substances released by one organism to enhance the development of another, and probiotics are known to be beneficial microbes that influence health through host immunomodulation and modulation of the bacteriome (8, 9). Several studies have found a positive association between the use of certain probiotic bacterial strains and oral health (10, 11), as well as those certain strains of lactobacilli, have anti-inflammatory capacity and reduce periodontal pathogens in the oral biofilm (12–14) and, precisely in this sense, have begun to be used for the treatment of peri-implantitis (15, 16). It has been shown that biofilms facilitate greater resistance to microorganisms, which translates into greater success in colonization and maintenance of the bacterial population (17), which is why certain biofilm-related infections, such as peri-implantitis, constitute an important clinical problem for the correct functioning and long-term survival of implanted devices (18).

A cause-effect relationship has been demonstrated between the accumulation of bacterial biofilms around titanium dental implants and the development of an inflammatory response (19) and that the clinical parameters evaluated returned to healthy levels after 21 days of biofilm control (20). However, biofilm control escapes both the immune system and the action of numerous antimicrobials, posing a danger to the survival of dental implants (21, 22) and that is, the use of probiotics and their derivatives has gained increasing interest in the fight against oral biofilms, with the aim of inhibiting their maturation and growth (23).

Biofilms together with the breakdown products of peri-implant bone tissue, in the case of peri-implantitis, appear to result in a local immune response in the infected tissue and the production of proinflammatory cytokines such as IL-1β, associated with the stimulation of fibroblasts, endothelial cells and osteoclasts (24). IL-6 also induces bone resorption and, moreover, acts synergistically together with IL-1β, and it has been shown that the levels of both cytokines in peri-implant gingivocrevicular fluid (PGF) are higher in areas with peri-implantitis than in healthy areas (25), although cytokine levels in healthy areas are not perfectly defined (26).

For all these reasons, the aim of the present systematic and meta-analytic approach of randomized clinical studies was to evaluate the efficacy of probiotics in the treatment of peri-implant oral diseases.

## 2 Methods

### 2.1 Study presentation and registration

This systematic review has been prepared according to “The Preferred Reporting Items for Systematic Reviews and Meta-Analyses (PRISMA) (27) and the guidelines of the Clinical Practice Guidelines (28). The protocol of this systematic review has been registered in INPLASY under the number: INPLASY202410051, doi number: 10.37766/inplasy2024.1.0051.

### 2.2 Question of interest, PICOs format

The focus of the research question was elaborated according to the PICOs format: “Are probiotics able to favorably modify clinical and immunological biomarkers determinants of peri-implant pathologies?”. Intervention studies in adult patients with mucositis, peri-implantitis or both (P) comparing probiotic treatment alone, or as adjuvant therapy (I) with patients receiving conventional treatment or placebo (C) were included to observe effects on clinical and immunological parameters (O), with only randomized clinical studies considered (**Table 1**).

**Table 1:** PICOs format.

Population	Adult subjects with mucositis, peri-implantitis, or both
Intervention	Probiotic treatment alone, or as adjunctive therapy
Comparisons	Conventional treatment or Placebo
Outcomes	Observe the effects of treatment on clinical parameters indicative of mucositis or peri-implantitis (Δ PD; Δ PI; Δ BoP) and/or immunological parameters (Δ IL-1β; Δ IL-6; Δ IL-8; TNF-α).
Study design	RCTs

PD, Probing Depth; PI, Plaque Index; BoP, Bleeding of Probing; IL-6, Interleukin-6; IL-1β, Interleukin-1-beta; TNF-α, Tumor Necrosis Factor alpha; RCTs, Randomized Controlled Trials.

PD, Probing Depth; PI, Plaque Index; BoP, Bleeding of Probing; IL-6, Interleukin-6; IL-1β, Interleukin-1-beta; TNF-α, Tumor Necrosis Factor alpha; RCTs, Randomized Controlled Trials.

### 2.3 Studies selection, eligibility criteria

The original research studies were selected according to the following inclusion criteria: (i) randomized clinical trials (single or double blind) that included in the study more than 10 subjects ≥ 18 years of age; (ii) with peri-implant pathologies; (iii) that provided data on clinical parameters and/or immunological follow-up indicative of peri-implant disease; (iv) with statistical methods that included mean numerical values and standard deviation, together with units with which to quantify mediator levels; (v) published in English. Studies that did not follow all the criteria defined above, with lack of relevant or demonstrative data on peri-implant disease, preclinical studies or *in vitro* studies, case series or clinical cases, literature reviews and irrelevant studies (editorial letters, conference abstracts, historical reviews…) were excluded.

### 2.4 Search approach

Two reviewers (NL-V, AL-V) independently searched four electronic databases (MEDLINE/PubMed, Embase, Cochrane Central, Web of Science) until December 2023, using the terms Medical Subject Headings (MeSH): Peri-Implantitis*/diagnosis OR Peri-Implantitis*/prevention & control OR Mucositis* AND Dental Implants* AND Dental Plaque* AND Probiotics/therapeutic use* AND Lactobacillus* AND Probiotics*/therapeutic use* AND Humans*. In addition, a manual search and consultations in the gray literature were performed; the bibliographic references of the included studies were also consulted to obtain the most information and avoid bibliographic bias.

### 2.5 Data collection

The data provided by each included study were extracted and tabulated by two reviewers (NL-V, AL-V) using the standardized data extraction tools of JBI-MAStARI. Both reviewers systematically screened the titles and abstracts of the previously selected studies. Those that met the inclusion criteria were extracted for reading and data extraction. Discrepancies between reviewers were resolved by discussion and judgment of a third reviewer (JABR). Cohen’s kappa index (κ) (29) was used to assess inter-reviewer agreement.

Because all included articles were randomized studies, the JBI-MAStARI Data Extraction Form for Randomized Controlled Trials was used. Data extracted from the studies included specific details about the interventions, populations, study methods, specific objectives, and significant outcomes to formulate the question of interest. All results were subjected to double data entry to minimize error bias.

### 2.6 Methodological rigor of the studies

The studies included in this systematic review and meta-analysis were methodologically evaluated using the tool developed by the Joanna Briggs Institute for RCTs (JBI MAStARI) which takes a particular view of the evidence and the methods used to synthesize the different types of evidence. The checklist consists of thirteen items and the responses to the items are “yes”, “no”, “unclear” or “not applicable”. The answer “yes” scores one point. To include a study, it had to score seven or more (30).

### 2.7 Risk of bias

Two assessors (NL-V and AL-V) independently assessed the risk of bias of the studies using the Cochrane Risk of Bias Tool (RoB2) (31) which assesses 7 domains of bias: Random sequence generation (Selection bias); Allocation concealment (Selection bias); Blinding of participants and staff (Performance bias); Blinding of outcome assessment (Detection bias); Incomplete outcome data (Attrition bias). Studies were assessed with “high”, “low” and “borderline” risk of bias; “borderline” risk of bias, was applied to those with a lack of information on possible bias. Discrepancies among the assessors were discussed to reach consensus.

### 2.8 Data synthesis

Data were analyzed using Review Manager software (RevMan Software. Version 5.4.1; The Cochrane Collaboration, Copenhagen, Denmark; 2020). Meta-analyses were performed for studies assessing peri-implantitis, mucositis and for different clinical and immunological variables, as well as a meta-analysis of pooled studies. All were based on mean difference (MD) and standard deviation (SD) to estimate continuous data and to evaluate categorical data, 95% confidence intervals (CI). Heterogeneity was considered unimportant with I^2 = ^0-30%; moderate, I^2 = ^40-50%; substantial I^2 = ^60-75% and considerable I^2^≥ 75%. The threshold for statistical significance was set as p < 0.05. Due to the homogeneity of results, a fixed-effects meta-analysis was performed.

## 3 Results

**Figure 1:**
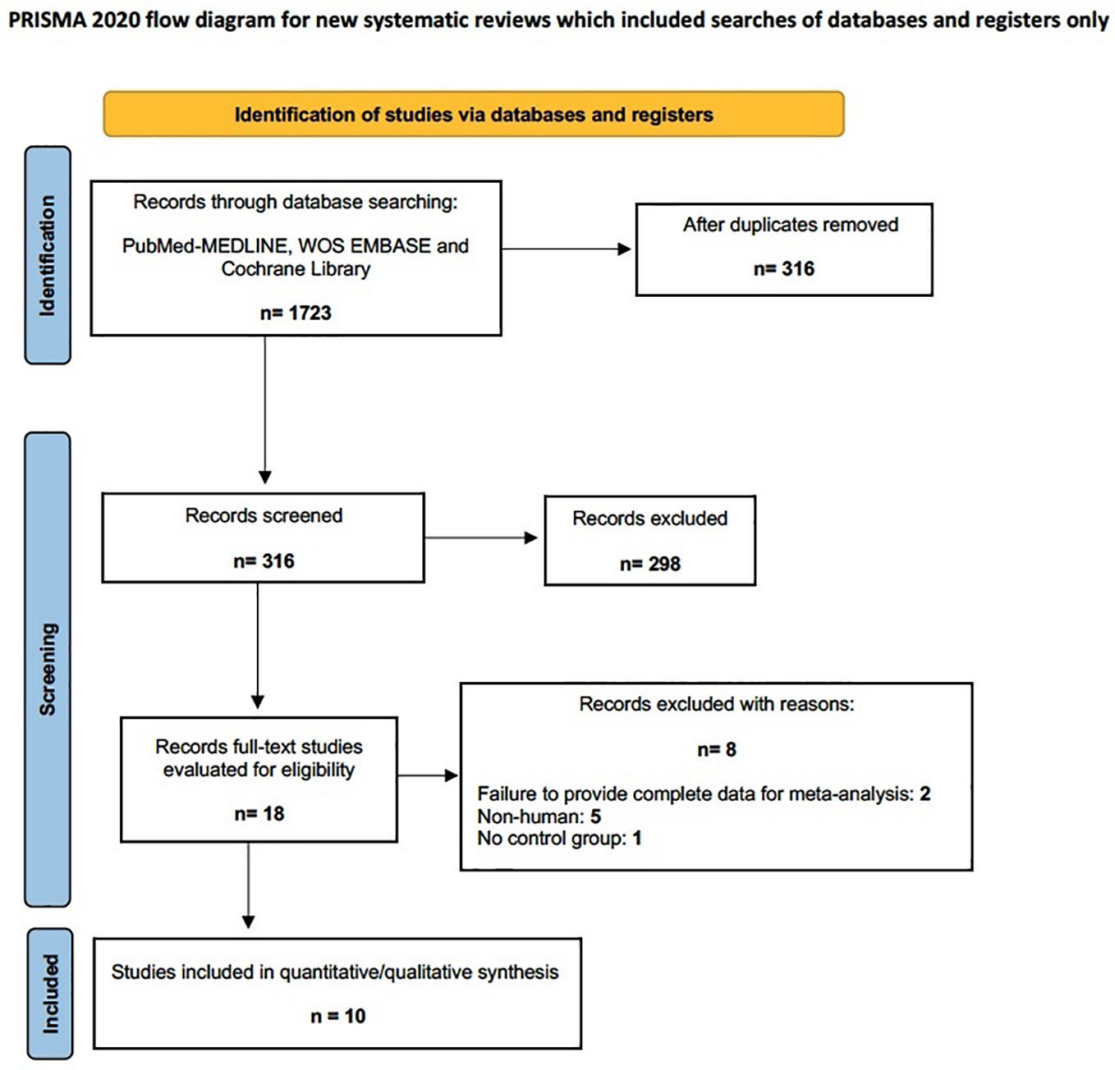
Flow diagram.

The electronic search found a total of 1723 results, which constituted 316 unique citations. Eighteen full-text publications were evaluated and 8 were excluded based on *a priori* criteria (**Figure 1**).

### 3.1 Qualitative analysis, characteristics of the studies

After exclusion, 10 studies were finally included in the meta-analysis (32–41). Inter-reviewer agreement when including studies exceeded 85% (κ > 85%).

A total of 340 patients and 238 implants were studied; however, 4 studies did not report the number of implants studied (33, 35, 37, 39). Follow-up periods ranged from 4 to 24 weeks. Two studies evaluated peri-implant pathology (35, 36) and only one (41) evaluated crestal bone loss (CBL). Regarding clinical evaluation, in the studies that assessed mucositis (32–34, 36–39), the clinical parameters PD and PI were evaluated in 5 studies (32, 33, 36, 38, 41) and BoP in 6 other studies (33, 36, 38–41). The most complete studies were those of Flichy-Fernandez et al. and Santana et al. (32, 39). Regarding immunologic evaluation, 3 studies evaluated cytokines IL-6, IL-8 and IL-1β (32, 33, 39) and only 2 studies evaluated TNF-α (33, 39). The study by Lauritano et al. (37) provided the least amount of analyzable data. The characteristics of the studies are shown in **Table 2**.

**Table 2:** Characteristics of studies and participants included in the systematic review.

Study, year	Type of study	Subjects number	Implants evaluated	Mucositis criteria	Peri-implantitis criteria	Follow-up	Probiotic type and dosage	Comparision	Detection method	Outcomes
Flichy-Fernández et al., 2015 (32)	Prospective, double-blind, placebo-controlled, crossover study.	34	77	PI; PD; GI; IL-6; IL-8; IL-1β		24 weeks	Lactobacillus reuteri. 1 tablet every 24 h	Placebo	Periodontal probing for bleeding. Visual method for detect plaque. For cytokine evaluation, Human Inflammation Cytometric Bead Array and cytofluorome analysis.	Reduction of 73.9% of mucositis in implants treated with probiotic.
Hallström et al., 2016 (33).	Double-blind randomized placebo-controlled trial.	49	NR	PD; BoP; PI; IL-1β; IL-4; IL-6; IL-8; TNF-α		4, 12, 26 weeks	Lactobacillus reuteri. Topical application sub and supragingivally of a drop of experimental oil with the Lactobacillus reuteri strains, around the selected implant.	Placebo	CGF using paper tips (Dentsply Maillefer size 55, Ballaigues, Switzerland) in the peri-implant pocket. The volume was recorded with a Periotrone 8000.	After 4 and 12 weeks PD and BoP were significantly reduced compared to baseline values.
Mongardini et al., 2017 (34)	Randomized, cross-over, placebo- controlled, double blind trial.	20	20	PI		6 weeks	Lactobacillus plantarum and Lactobacillus brevis. Probiotic mixture prepared with saline solution and probiotic powder in a 1:1 ratio in the peri-implant sulcus.	Placebo	BoP was assessed using a pressure-sensitive hand-held periodontal probe (UNC15; Hu Friedy, Milan, Italy) with a force of approximately 0.2 N. Cytokine sampling using a commercial Bio-Plex Cytokine Assay.	No significant difference in clinical outcomes was observed between treatment groups.
Tada et al., 2017 (35)	Randomized placebo-controlled clinical study.	30	NR		PD; BoP; PI	4, 12, 24 weeks	Lactobacillus reuteri. 1 tablet every 24 h	Placebo	For PD, a soft plastic periodontal probe for implants with torque control at 0.25 N was used. BoP was assessed at 6 sites along the mucosal margin adjacent to the implant. Plaque was recognized by passing a probe over the smooth marginal surface of the implant.	PD in the probiotic group was significantly lower at 4 and 24 weeks than at baseline. No significant differences in BoP were observed between the groups.
Gallofré et al., 2018 (36)	Randomized, controlled, parallel-design, triple-blind prospec- tive clinical study.	44	44	PD; BoP; PI	PD; BoP; PI	12 weeks	Lactobacillus reuteri. 1 tablet every 24 h	Placebo	PD at implant level was recorded in millimeters using a periodontal probe with a force of 0.2-0.3N. IP was recorded according to O’Leary et al. BoP according to Ainamo and Bay.	The probiotic together with mechanical therapy produced an additional improvement over treatment with mechanical therapy alone, both in bleeding on probing and in probing pocket depth.
Lauritano et al., 2019 (37)	Randomized clinical trial	10	NR		GI	4 weeks	Lactobacillus reuteri. 1 tablet every 24 h	Placebo	NR	No significant differences between groups
Peña et al.2019 (38)	Triple-blind parallel randomized clinical trial	50	50	PD; PI; BoP;		12 weeks	Lactobacillus reuteri. 1 tablet after brushing your teeth in the evening	Placebo	PD and BoP were assessed with a periodontal probe at six sites per implant.	After probiotic or placebo administration, clinical variables, except PD, increased slightly and progressively until the 3-month follow-up, but without reaching baseline levels.
Santana et al., 2022 (39)	Randomized placebo-controlled clinical trial	36	NR	PD; BoP; IL-1β; IL-6; IL-8; TNF-α		12, 24 weeks	Lactobacillus rhamnosus, Lactobacillus paracasei and Bifidobacterium animalis. Topical gel application	Placebo	PD and BoP was evaluated using a plastic probe.	PI and PD experienced no significant differences between the test and control groups. Both groups presented a reduction in BoP at 12 and 24 weeks. At 24 weeks, only the experimental group presented lower levels of IL-1β, IL-6, IL-8 and TNF-α than at baseline.
Sargolzaei et al., 2022 (40)	Double-blind randomized controlled trial	25	47	PD; BoP		4 weeks	Lactobacillus. Nightly rinse with 1 probiotic capsule dissolved in 2 tablespoons of warm water.	Placebo	Peri-implant probing.	Efficacy of the probiotic in improving BoP in the short term, but no significant effect on PD.
Alqahtani et al., 2022 (41)	Parallel arm trial	42	NR	PD; PI; BoP	CBL	12, 24 weeks	Nonsurgical-mechanical debridement + Lactobacillus Reuteri. 1 tablet every 12 hours after brushing teeth.	Nonsurgical-mechanical debridement alone	Peri-implant PD was measured using a graduated plastic probe.	In the control group, PI, BoP and PD were comparable with the respective baseline values at 6-month follow-up. CBL in all groups remained unchanged until 6 months follow-up.

PI, Plaque Index; PD, Probing Depth; GI, Gingival Index; BoP, Bleeding of probing; CGF, Crevicular Gingival Fluid; CBL, Crestal Bone Loss; IL-4, Interleukin-4; IL-6, Interleukin-6; IL-8, Interleukin.8; IL-1β, Interleukin-1-beta; TNF-α, Tumor Necrosis Factor alpha; NR, No Report.

### 3.2 Assessment of methodological rigor

The methodological quality of all included studies ranged from high (10 points) to very high (>10 points), as determined by the JBI-MAStARI critical appraisal checklist for RCTs. Only two studies (37, 40) were not evaluated due to missing data. (**Table 3**).

**Table 3:** Results of critical appraisal for Randomized Controlled Trials (JBI MAStARI).

Study	Q1	Q2	Q3	Q4	Q5	Q6	Q7	Q8	Q9	Q10	Q11	Q12	Q13	Total Score
Flichy-Fernández et al., 2015 (32)	1	1	1	1	1	0	1	1	1	1	1	1	1	12
Hallström et al., 2016 (33).	1	1	1	1	1	0	1	1	1	1	1	1	1	12
Mongardini et al., 2017 (34)	1	1	1	1	1	1	1	1	1	1	1	1	1	13
Tada et al., 2017 (35)	1	1	1	1	?	1	1	1	1	1	1	1	1	12
Gallofré et al., 2018 (36)	1	1	1	1	1	1	1	1	1	1	1	1	1	13
Lauritano et al., 2019 (37)	?	?	?	?	?	?	?	?	?	?	?	?	?	0 No data reported
Peña et al.2019 (38)	1	1	1	1	1	1	1	1	1	1	1	1	1	13
Santana et al., 2022 (39)	1	1	1	1	1	?	1	1	1	1	1	1	1	12
Sargolzaei et al., 2022 (40)	?	?	?	?	?	?	?	?	?	?	?	?	?	0 No data reported
Alqahtani et al., 2022 (41)	1	1	1	1	1	1	1	1	1	1	1	1	1	13

Q1. Was true randomisation used for assigning participants to treatment groups?; Q2. Was allocation to treatment groups concealed?; Q3. Were treatment groups similar at the baseline?; Q4. Were participants blind to treatment assignment?; Q5. Were those delivering treatment blind to treatment assignment?; Q6. Were outcomes assessors blind to treatment assignment?; Q7. Were treatment groups treated identically other than the intervention of interest?; Q8. Was follow-up complete and if not, were differences between groups in terms of their follow- up adequately described and analysed?; Q9. Were participants analysed in the groups to which they were randomized?; Q10. Were outcomes measured in the same way for treatment groups?; Q11. Were outcomes measured in a reliable way?; Q12. Was appropriate statistical analysis used?; Q13. Was the trial design appropriate, and any deviation from the standard RCT design accounted for in the conduct and analysis of the trial?.

### 3.3 Meta-analysis, risk of bias

For the meta-analysis, only 9 studies were used, as the study by Lauritano et al. (37) was not used because it did not provide analyzable data.

GI variables in mucositis and CBL in peri-implantitis, were not analyzed in the meta-analysis because they were evaluated by only one study each [32 and 41, respectively].

Two meta-analyses were performed, one for studies evaluating probiotics in mucositis and one for studies evaluating probiotics in peri-implantitis. Clinical and immunological parameters and an analysis of grouped variables in both cases (mucositis and peri-implantitis) were analyzed independently. All subgroups were homogeneous (I^2 = ^0%), except in the analysis of IL-6 in mucositis (32–34, 39), which resulted in substantial heterogeneity (I^2 = ^65%). In both mucositis and peri-implantitis, the overall effect was in favor of the experimental group. Although not statistically significant, the analysis of studies grouped in peri-implantitis showed a trend towards significance (p=0.09).

No adverse effect analysis was performed due to lack of data.

**Figure 2:**
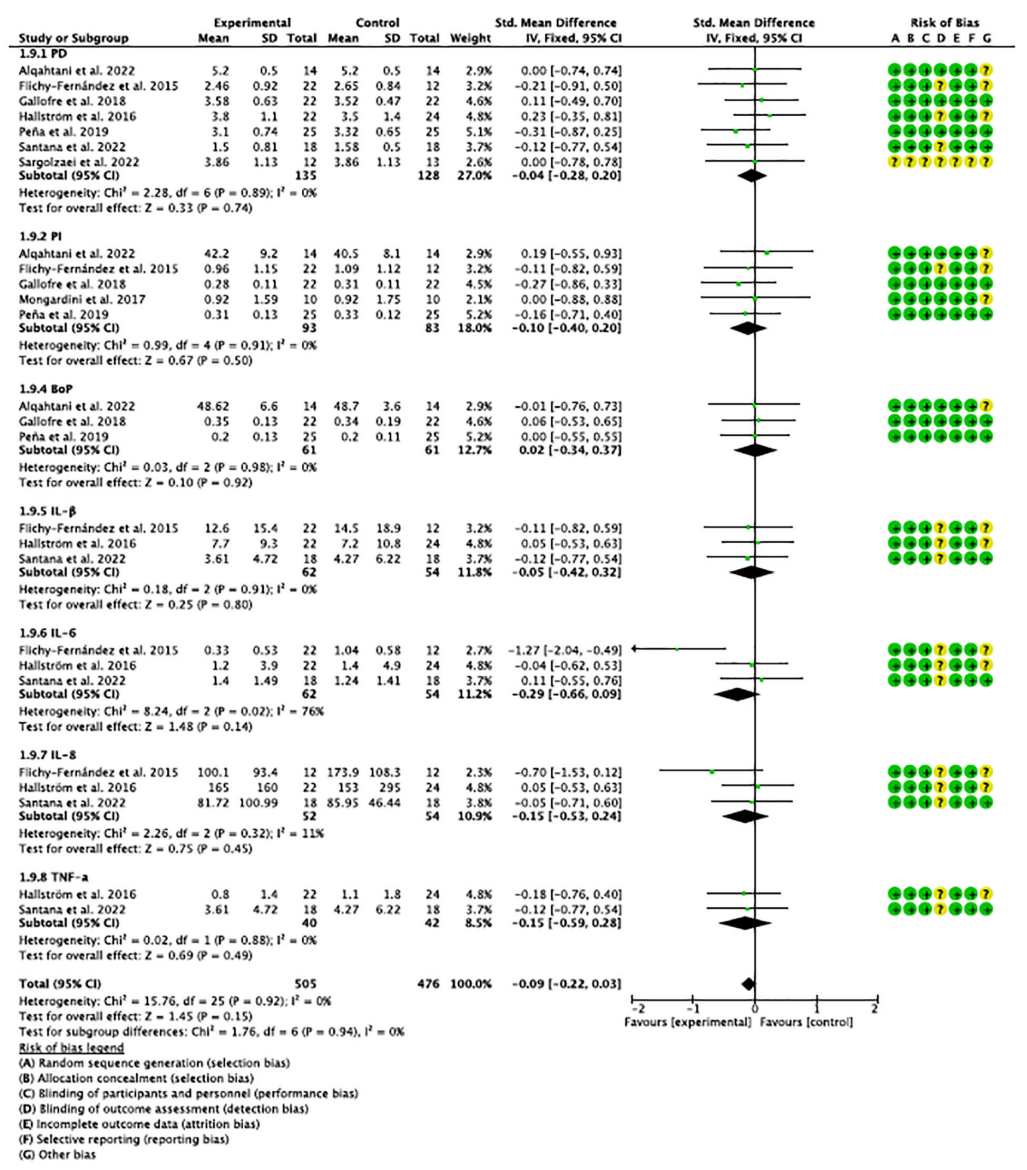
Forest plot and risk of bias (on the side) of studies evaluating clinical and immunological parameters in mucositis.

**Figure 3:**
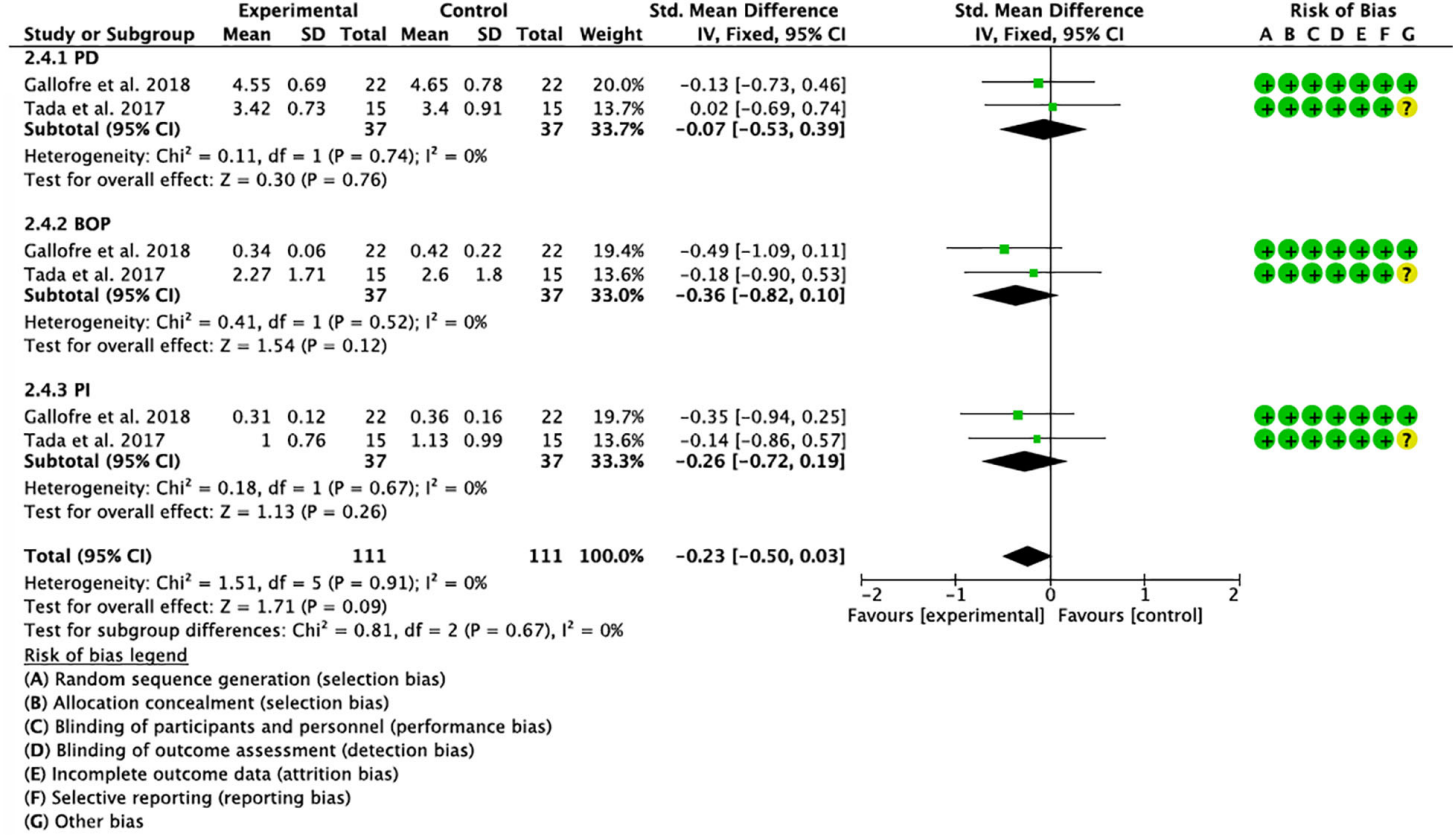
Forest plot and risk of bias (on the side) of studies evaluating clinical parameters in peri-implantitis.

Risk of bias assessment is one of the pillars of evidence-based medicine; therefore, two reviewers (NL-V and AL-V) independently analyzed the quality of the included studies according to the Cochrane Risk of Bias tool (42). Disagreements between reviewers were resolved by consensus or discussion. The tool evaluates randomized studies in 5 domains: (1) the randomization process; (2) deviations from the intended interventions; (3) missing outcome data; (4) outcome measurement; and (5) selection of the reported outcome and a sixth bias relating to (6) other biases. According to the Cochrane Handbook for Systematic Reviews of Interventions, a “high” rating was given to studies considered at high risk of bias, “low” to those at low risk of bias, and “borderline” to those with uncertain bias or lack of information on potential bias. However, some studies included randomization software, and it was difficult for the reviewers to know which domains they addressed, so most reported uncertainty in the “other biases” domain. Nevertheless, the studies considered met most of the domains and were considered to have a low risk of bias, except for the study by Sargolzaei et al. (40), which did not provide evaluable data. (**Figures 2**, **3**).

### 3.4 Publication bias

**Figure 4:**
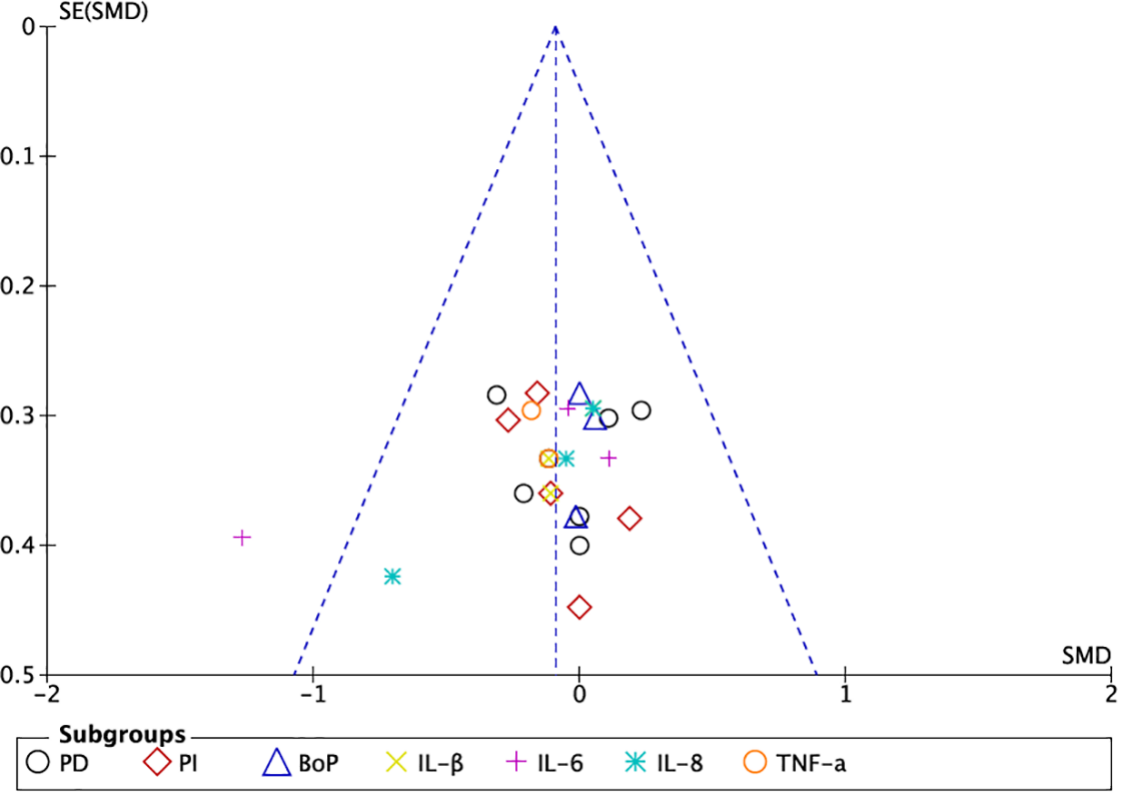
Funnel plot of grouped studies in mucositis.

**Figure 5:**
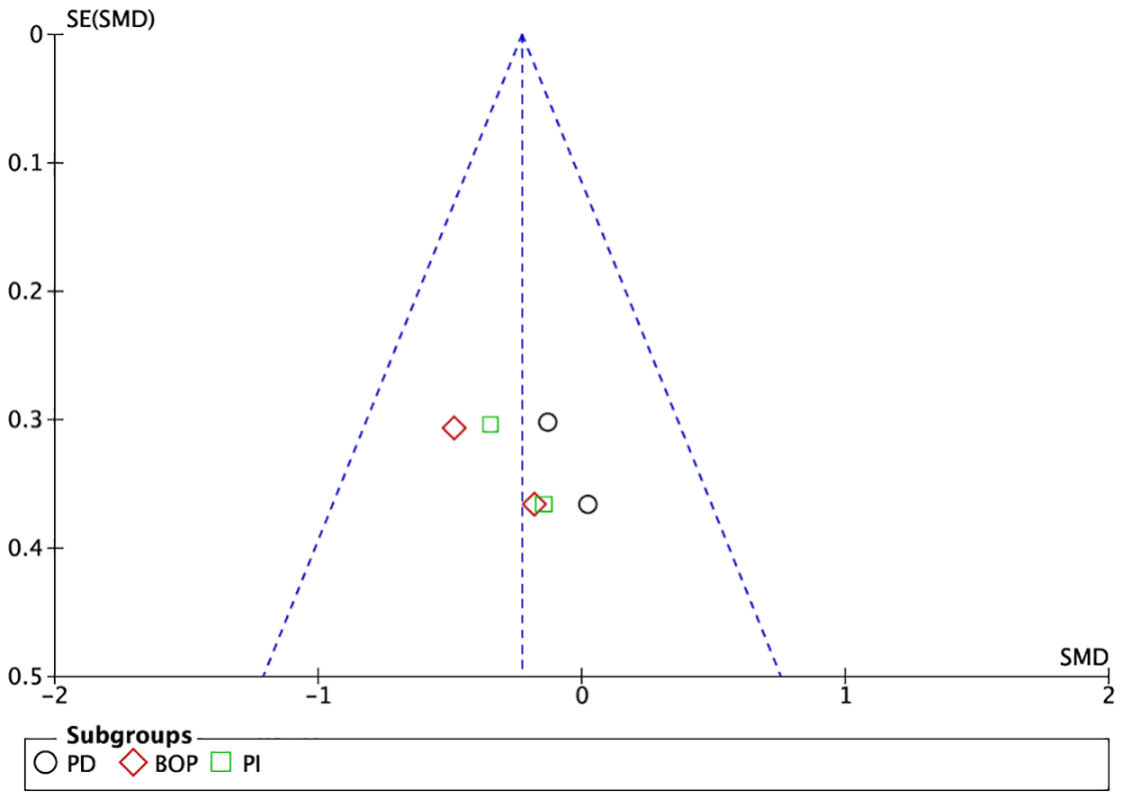
Funnel plot of grouped studies in peri-implantitis.

The graphs in **Figures 4**, **5**, in which the abscissa axis (X) represents the observed results and the ordinate axis (Y) the standard error, show a low asymmetry and, therefore, a low publication bias.

## 4 Discussion

### 4.1 General discussion of results

Nine clinical trials were included in our meta-analysis (32–36, 38–41) and all of them reported beneficial effects of probiotics in the treatment of peri-implant diseases, both for clinical and immunological parameters. Therefore, and taking into consideration the limitations of conventional treatment to solve peri-implant pathologies and especially the inflammatory process, it is interesting to look for therapeutic alternatives, either alone or as adjuvants.

Traditionally, different methods have been applied for the prevention and treatment of peri-implantitis, such as mechanical debridement, surgical therapy with or without regenerative procedures and antibiotics, either local or systemic (43), but it is known that microbial dysbiosis generates inflammatory processes that are difficult to resolve, due to alterations in the immunoinflammatory response of the host; therefore, new treatments that are able to restore the balance of the microbiome and give rise to a healthy oral microbiome are currently being investigated (44). A Cochrane systematic review by Esposito et al. (45) reported that more complex and expensive therapies for the treatment of peri-implantitis were not more beneficial than control therapies, suggesting that, being a chronic pathology, periodic repetition of treatments may be necessary, recommending well-designed RCTs with follow-ups longer than 12 months. Iniesta et al. (46), in a systematic review and meta-analysis demonstrated the beneficial effect of probiotics on clinical inflammatory parameters. In this aspect, our meta-analysis, despite finding an overall favorable effect in the experimental group, did not find statistical significance in any of the groups; only the analysis of studies grouped in peri-implantitis showed a tendency towards significance.

One aspect to take into consideration is the current surfaces of dental implants, which are rougher in order to seek greater bone-implant contact. It has been found that this type of surface, unlike smooth surfaces, in addition to being conducive to the formation of biofilms and bacterial accumulation, makes cleaning difficult when exposed, facilitating peri-implant diseases, although there is no unanimity on these criteria (47, 48). Disruption of host-microbe homeostasis at the implant-mucosa interface caused by biofilm accumulation leads to peri-implant mucositis and optimal removal of biofilm is considered a prerequisite for the prevention and treatment of peri-implant mucositis, a precursor of peri-implantitis (49).

Biomarkers objectively assess biological processes, normal, pathological or in response to a given intervention (50) and are instrumental in the diagnosis and monitoring of peri-implant diseases (51).The cells of the gingival epithelium, fibroblasts, neutrophils and macrophages, release cytokines, such as IL-6, IL-1β and NFα, which cause the degradation of connective tissue and alveolar bone, and about 100 different molecular components have been evaluated in the diagnosis of potential periodontal diseases, although for the diagnosis of peri-implantitis, this number is reduced to half. A recent review by Pliavga et al. (52) involving 1,117 patients with 1,346 implants revealed 49 different biomarkers, with significantly higher values of IL-1β, IL-6 and TNF-α levels in the group of subjects suffering from peri-implantitis. In our meta-analysis, 3 studies (32, 33, 39) evaluated three interleukins (IL-1β, IL-6, IL-8) and only two studies (33, 39) evaluated TNF-α, as indicative of mucositis; however, none of the studies included in our meta-analysis evaluated interleukins as determinants of peri-implantitis, limiting the results, exclusively to clinical parameters. Flichy-Fernandez et al. (32) found statistically significant decreases in IL-1β, IL-6 and IL-8 after probiotic administration; Hallström et al. (33) only found statistical significance in IL-8 and TNF-α values and Santana et al. (39), found lower levels of IL-1β, IL-6, IL-8 and TNF-α, in the group of subjects with mucositis treated with probiotics, only, 24 weeks after the start of the study, finding no differences in shorter time periods.

The effect of probiotics on clinical parameters in peri-implant pathology was evaluated in 8 studies on mucositis (32–34, 36, 38–41) and 2 on peri-implantitis (35, 36), of those included in our meta-analysis.However, the objectives of the studies included after the use of probiotics were discrepant: While most of them studied their effects on mucositis, Tada et al. (35) studied their action on peri-implantitis and Gallofré et al. (36), on both pathologies in patients with associated periodontal pathology. On the other hand, the different studies used different probiotic treatments, dosages and routes of administration. Most of the studies used *Lactobacillus reuteri* and oral administration; however, Sargolzaei et al. (40) used topical local application and Mongardini et al. (34) combined local and systemic use.

There is controversy about the diagnostic thresholds of peri-implant diseases and about the validity and diagnostic evidence of PD and BoP which, despite having traditionally been clinical markers of periodontal diseases, are not considered as such in peri-implant diseases. The same criterion prevails for CBL and there is no unanimity on whether these parameters could be related to the diagnosis of peri-implant disease (53). One of the studies included in our meta-analysis (41) compared mesial and distal CBL in subjects with adjunctive probiotic treatment, with mechanical debridement alone, finding no differences between groups; however, studies on mucositis (32, 33, 36, 38–41) reported discrepant results for the experimental groups versus placebo: while Flichy-Fernandez et al., Hallström et al., Gallofré et al. (32, 33, 36) reported statistically significant results in decreasing PD, Peña et al., Santana et al., Sargolzaei et al., and Alqahtani et al. (38–41) observed no significant differences between experimental and control groups. Similarly, Tada et al. and Gallofré et al. (35, 36) disagreed on PD measures in peri-implantitis, the former finding no significant differences between the probiotic and placebo groups. We also found discrepant results in studies investigating BoP. Gallofré et al. and Peña et al. (36, 38) reported improvements in bleeding on probing for the probiotic-treated group, both in implants with mucositis and periodontitis, while Santana et al. and Alqahtani et al. (39, 41) found no differences between groups.

In this context of validation of clinical parameters as determinants of peri-implant diseases, these parameters are questioned as indicative of such pathologies. It has been shown that PD and peri-implant soft tissue thickness, is greater in implants than in adjacent teeth (54) and in this regard Doornewaard et al. (53) in a critical review, pointed out a lack of correspondence between PD and BoP values and peri-implant disease.

On the other hand, some studies have shown a genetic disposition on the part of certain subjects to suffer this type of pathology. Cardoso et al. (24) in a pilot study that included a sample of 20 subjects, found a possible relationship between the IL-1 genotype and peri-implantitis, although another larger study on 98 subjects found no differences in the IL-8 polymorphism between groups with peri-implantitis and healthy subjects (55).

Non-surgical treatments of peri-implant diseases include mechanical debridement, adjunctive antiseptic therapy, antibiotic therapy, laser-assisted therapy, adjunctive treatments with natural products and other combined approaches (56), however, in addition to biological parameters, immunoinflammatory mediators have to be considered by clinicians to determine peri-implant health and disease states. In addition, genetic data could contribute to predict the prognosis of treatments.

### 4.2 Considerations and limitations

The systematic review is essentially an analysis of the evidence in the available scientific literature and a judgment on the efficacy of a treatment, which involves a series of complex steps that can give rise to biases and limitations.

We highlight the latter: the different pathologies analyzed (mucositis and peri-implantitis), with disparate populations and follow-ups and equally different sociodemographic conditions; the different types of probiotic strains used by the studies, their application and comparison; the statistical analyses of the included studies, despite being evaluated as adequate, are unequal. All this biases and may alter the reporting of results. Finally, it should be noted that the Hawthorne effect, which causes behavioral changes in individuals that are observed in epidemiological studies, was only taken into account in the study by Flichy-Fernandez et al. (32) and was not considered in the other included studies.

## 5 Conclusions

The use of probiotics, either as a basic or complementary treatment of peri-implant diseases, showed a trend towards statistical significance and in view of the increasing demand for alternative natural treatments, they could prove to be a complementary method of treatment of peri-implant diseases.

Well-designed randomized studies are justified and necessary to validate the efficacy of these products in peri-implant pathologies.

## Data availability statement

The original contributions presented in the study are included in the article/supplementary material. Further inquiries can be directed to the corresponding author.

## Author contributions

NL-V: Conceptualization, Formal analysis, Methodology, Writing – original draft, Writing – review & editing. AL-V: Project administration, Writing – original draft, Writing – review & editing. JABR: Supervision, Writing – original draft, Writing – review & editing.
